# A pilot study to understand faculty and student needs for undergraduate medical education simulation sequencing in the United Arab Emirates

**DOI:** 10.15694/mep.2021.000127.1

**Published:** 2021-05-14

**Authors:** Dima Abdelmannan

**Affiliations:** 1 Dubai Health Authority

**Keywords:** Undergraduate curriculum design, Simulation curriculum design, Curriculum sequencing, Prerequisite knowledge, Outcome-based curriculum, Backward design

## Abstract

This article was migrated. The article was marked as recommended.

**Introduction:** The use of simulation-based education in undergraduate medical education has many advantages. Purposeful planning of the sequence of simulation sessions within the curriculum is necessary for complex simulation exercises. This requires pre-session completion of prerequisite knowledge.

**Methods:** Two surveys were conducted. An electronic survey was sent to faculty involved in simulation at UAE medical schools (n=29). The faculty survey addressed the simulation sessions planning process, sequencing of simulation topics, and institutional simulation team structure. The second survey was administered via paper to final year medical students from Dubai Medical College (DMC) who received simulation sessions (n=22). The survey addressed completion of prerequisite knowledge, receipt of the session objectives, and psychological safety and overall session ratings. Quantitative data were analyzed descriptively. Responses to open ended questions were analyzed thematically.

**Results:** The faculty response rate was (21/29: 72.4%). Only (7/21: 33.3%) faculty members indicated there was prior planning to ensure proper sequencing within the curriculum. Only (3/21: 14.2%) indicated that simulation topics were chosen based on curriculum gaps. A small percentage (3/21: 14.2%) reported working with other faculty and an instructional designer. Qualitative themes included barriers to planning, structural considerations, and factors for successful simulation integration.

The student response rate was 100%. Only (4/22:18.2%) of students completed the required pre-session prerequisite knowledge. Most students (18/22: 81.8%) received the session objectives prior to the simulation session. Qualitative themes included lack of completion of pre-requisite knowledge prior to the session, absence of learning objectives, and technical issues.

**Conclusion:** This study highlights the gap in adequate sequencing of complex simulation scenarios within the curriculum. This is mainly due to the lack of completion of the required prerequisite knowledge prior to the session. Such complex integration requires adequate planning and collaboration of the simulation team with key stakeholders including faculty, the curriculum committee, and instructional designers.

## Introduction

The use of simulation in medical education has gained momentum ( Passiment, Huang and Sacks, 2011). Between 84-94% of United States medical schools use simulation during each of the four years of undergraduate medical education ( Passiment, Huang and Sacks, 2011). In the United Arab Emirates (UAE), simulation use in teaching is also expanding across medical schools and allied health professions including nursing and pharmacy.Institutions in general vary in the way in which simulation technology is utilized, how instructors are engaged, and the extent to which simulation-based education is incorporated into the curriculum ( Cook, Friedman and Greene, 2007; La Cerra *et al.,* 2019; Kane-Gill and Smithburger, 2011).

Simulation has many advantages including improving clinical judgement and quality of graduates without compromising patient safety ( Aebersold, 2018). For the learner, simulation provides a safe environment during more complex roles which involve higher levels of critical thinking and clinical judgment ( Aebersold, 2018). It also allows active engagement with fellow learners while connecting with instructional material ( Stefaniak and Turkelson, 2014). Simulation modalities range from low technology models used for skill training and screen-based computer simulators to more complex task trainers, standardized patients, and ultimately high-fidelity replication of multiple and high-risk clinical conditions. As the modality becomes more sophisticated, simulation-based exercises become more complex, resembling real life clinical situations (e.g., conducting CPR for an unresponsive patient, managing airways, or recalling the name, dose, and route of a medication depending on the situation). Such environment allows the learner to practice problem-solving and decision-making skills, interpersonal and communications skills, and team-based competencies at the same time as in real life. At the same time, it requires the learner to possess a baseline amount of knowledge and skills.This is known as “prerequisite knowledge” or “enabling objectives.” If the prerequisite knowledge is acquired, completed, and practiced prior to the session, it allows the learner to be aware and respond appropriately ( Fraser, Ayres and Sweller, 2015; Stefaniak and Turkelson, 2014).

Another illustration of the above can be elaborated using the cognitive load theory model described by ( Young *et al.,* 2014) which builds upon the human memory model that includes the subsystems of sensory, working, and long-term memory. Working memory (the part of short-term memory which is concerned with immediate conscious perceptual and linguistic processing) can only process limited elements of information at any given time. In the simulation examples given above, unless the required tasks are mastered previously, they will exceed the working memory capacity of the learner and lead to impairment of the performance and the learning. In other words, the learner’s prior knowledge determines the ease with which the current information can be processed in working memory without causing cognitive overload.Another important factor that affects learner performance in such conditions is the emotional state of the learner ( Fraser, Ayres and Sweller, 2015). Despite ensuring the psychological safety of the environment in simulation-based education, having to respond to high stake conditions like a deteriorating patient or an emergency will lead to increase self-consciousness and anxiety which also contributes to consuming working memory resources ( Leppink and van den Heuvel, 2015; Simon, Raemer, and Rudolph, 2010). The above highlights the importance of completion of prerequisite knowledge prior to the simulation-based activity. In general, the less prior knowledge a learner has, the more prone they are to suffer from cognitive overload ( Fraser, Ayres and Sweller, 2015).If a topic is too complex to assimilate, the learning will not happen in one setting. This might happen several times until insights are gradually gained, understanding is achieved, and the subject material is appreciated and learned.

Yet, there is little understanding of the best approach to sequence and plan simulation sessions within the undergraduate medical curriculum. Few studies have examined curriculum sequencing in relation to the use of simulation, with variable results ( Huwendiek *et al.* 2013; Leon, Hajjar and DeSevo Bellottie, 2015; Stefaniak and Turkelson, 2014; Woda *et al.,* 2016). Some studies showed that learners who participated in simulation before lecture demonstrated increased knowledge compared with learners who participated in simulation after a lecture ( Stefaniak and Turkelson, 2014) and that students preferred integrated simulation sessions into the curriculum to a non-integrated approach ( Huwendiek *et al.,* 2013). Other studies showed that there were no significant differences in student performance on assessments whether they covered the simulation curriculum followed by in-clinic experience or vice versa ( Leon, Hajjar and DeSevo Bellottie, 2015) and that even when students preferred having simulation-based activities prior to hospital placements, the sequence did not impact their clinical decision making ( Woda *et al.,* 2016).

There have been no studies that looked specifically at sequencing in terms of completing the pre-requisite knowledge and the best way to achieve that with more complex scenarios. Given that simulation in medical education is relatively new, simulation experts tend to be a minority in relation to the whole pool of faculty in the healthcare environment ( Ladkany and Pastorino, 2020). This, along with the fact that students complete their rotations in blocks at different times, may lead to lack of completing the enabling objectives prior to the simulation session.

At the Dubai Medical College (UAE), complex simulation scenarios using high fidelity mannequins were introduced as a tool for enabling students to practice clinical reasoning and decision-making skills in a simulated team environment. Over time, feedback from faculty, simulation technicians, and students indicated that prerequisite knowledge and clinical skills in the simulation sessions had not yet been delivered and mastered prior to the session even though the general session topic aligned with the curriculum. Thus, we endeavored to understand the perceptions of both faculty and students about the extent of covering the enabling objectives prior to simulations sessions.

## Methods

As both faculty and students are key stakeholders, surveys were conducted among both groups. The anonymous faculty survey was an online cross sectional needs assessment survey, sent to faculty involved in simulation across all six UAE medical schools. The purpose was to understand whether there were similarities among current simulation practices. Inclusion criteria included faculty teaching with simulation in an undergraduate medical school program in the UAE. Faculty were identified by the author through a professional online group for UAE simulation educators where the link to the survey was posted (n= 25). The survey was also sent to the medical school Deans with a request to forward it to their institution’s simulation team. Four additional faculty were identified through this approach making the total number of eligible faculty 29.

Faculty were asked to express freely any perceived barriers to sequence planning and integration of simulation session within the curriculum and what can be done to improve sequencing of simulation within the curriculum.

The student survey was administered to final year medical students from Dubai Medical College who were enrolled in simulation-based rotation block. The simulation sessions were conducted using scenarios prepared by faculty conducting the sessionsusing high fidelity mannequins. Five topics were covered including shortness of breath, pneumonia, sepsis, acute myocardial infarction, and loss of consciousness. The session objectives and a brief scenario were emailed to the students one week before the session.

The paper survey was distributed to students at the end of the simulation session. The survey addressed three main areas 1) Completion of prerequisite knowledge prior to the session; 2) Receiving the session objectives and 3) Psychological safety and overall session rating.

This study was approved by the Dubai Medical College Ethics Committee.

## Results/Analysis

### Data Analysis

Data were analyzed descriptively. Quantitative data were presented as count and percentage of the respondents which answered “yes” for each question. SPSS 23 was used to calculate counts and percentages ( *IBM Corp (2015)*). Responses to open ended questions were analyzed thematically. After they were collated and reviewed to gain understanding and familiarity with the data, recurring messages were identified, and codes generated in the form of phrases to represent significant findings. The codes aimed to identify elements faculty and students identified as important to understanding curriculum sequencing.

### Results

**Faculty survey results ( Table 1):** (21/29: 72.4%) of faculty members responded. One third of responders (7/21: 33.3%) indicated there was intentional planning of the sequence of simulation sessions ensuring prerequisite knowledge and enabling objectives are met. One third (7/21: 33.3%) worked with other faculty or team members to plan sessions to ensure alignment of objectives while (8/21: 38.1%) worked with their curriculum committee. Regarding simulation topic choice and design, a minority of the faculty (3/21: 14.3%) indicated they chose topics based on gaps in curricular blueprint, and only (3/21: 14.3%) worked with an instructional designer. Regarding the simulation team (2/21: 9.52%) of faculty members were the only simulation champions at their institutions while (14/21: 66.6%) had few simulation faculty, and (5/21: 23.8%) had a full simulation team. Nearly half of respondents (9/21: 42.8%) reported having leadership support for simulation initiatives.

Three common themes were revealed through our thematic analysis: 1) Barriers to simulation planning; 2) Characteristics of simulation team structure and organization, and 3) Factors for successful integration of simulation into the curriculum. Barriers to simulation planning included working in isolation from the clinical teams, faculty of didactic course, and curriculum committee as well as lack of detailed mapping of the curriculum to ensure coverage of prerequisite knowledge. Structural factors were related to lack of awareness of both institution and faculty members of simulation team efforts and workflow. Successful integration factors included ensuring collaboration between stakeholders mainly curriculum committee and faculty including clerkship directors as well as working with an instructional designer consistently.

**Student survey results ( Table 2):** All students (22/22: 100%) completed the post-simulation session survey. Only two students (2/22: 9.1%) completed the necessary pre-requisite knowledge prior to the simulation sessions. Four students (4/22: 18.2%) did not receive the learning objectives prior to the simulation session. Two students (2/22: 9.1%) faced technical issues during the session. More than half of students (13/22: 59.1%,) rated the session as “excellent.”

Three common themes were revealed through our thematic analysis: 1) Lack of completion of prerequisite knowledge and enabling objectives prior to the simulation; 2) Not receiving learning objectives for the simulation session; and 3) Technical issues during the session. Lack of prerequisite knowledge was in the form of either incompletion of certain didactics or procedures prior to the session. Those came up during the simulation session and caused learner overload. Lack of learning objectives was due to not receiving of the session objectives on time. Technical issues were mainly due to inability to hear the audio during the session.

**Table 1:** Faculty survey results

Faculty Survey	Response (Yes)
**Sequence planning of simulation topics**	
1. Ensured prerequisite knowledge/ enabling objectives are met	(7/21: 33.3%)
2. Worked with other faculty members (clinicians and didactic lecture faculty) on planning sessions	(7/21: 33.3%)
3. Worked with curriculum committee to ensure correct sequence of topics prior to simulation	(8/21: 38.1%)
4. Chose topics based on gaps and blueprint	(3/21: 14.3%)
5. Worked with instructional designer	(3/21: 14.3%)
**Simulation team structure**	
1. Only simulation champion	(2/21: 9.52%)
2. Few simulation faculty	(14/21: 66.6%)
3. Full simulation team	(5/21: 23.8%)
4. Leadership support of simulation and simulation team	(9/21: 42.8%)
**Qualitative themes**	
1) Barriers in planning for simulation sessions	Work in isolation from the clinical teams, faculty of didactic course, and curriculum committee: “I would like to see more faculty from the clinical teams work with us” Lack of detailed mapping of the curriculum to ensure coverage of prerequisite knowledge: “We are not always aware of the topics covered prior to the session”
2) Structural and institutional considerations	Lack of awareness of both institution and faculty members of simulation team structure and workflow: “More departments can use our facilities”
3) Factors for successful integration of simulation sessions into the curriculum	Ensure collaboration between stakeholders mainly curriculum committee and faculty including clerkship directors: “Simulation sessions will be more useful if we worked with the other faculty and curriculum committee” Work with instructional design consistently: “Working with instructional designer - who we lost recently - was helpful in designing the scenarios”

**Table 2:** Student survey results

Student Survey	Response (Yes)
1. Prerequisite knowledge received prior to session	(2/22: 9.1%)
2. Objectives received prior to session	(18/22: 81.8%)
**Overall session rating (1-7)**	
5 (average)	(2/22: 9.9%)
6 (excellent)	(13/22: 59.1%)
7 (outstanding)	(7/22: 31.8%)
**Qualitative themes**	
1) Enabling objectives not acquired prior to the session	Lack of coverage of necessary topics prior to the simulation session: “Our group didn’t complete the didactics on septic shocks and this case required that” Lack of coverage of necessary procedures prior to the simulation session “If I have practiced airway management, I would have known how to handle the case better”
2) Session objectives not received prior to the session.	Lack of clear objectives for the simulation session: “Our group didn’t receive the objectives for the session”
3) Technical issues during the session	Audio issues with the mannequin: “I couldn’t hear clearly what the patient was saying”

## Discussion

This study explored the perceptions of faculty and students towards the process of sequencing of simulation within the undergraduate medical education curriculum. The goal was to assess the experiences and processes of faculty and students on pre-session planning to ensure proper sequencing by completion of prerequisite knowledge prior to the sessions.

Both faculty and students’ responses demonstrated a gap in the planning process and completion of prerequisite knowledge prior to the sessions. Faculty responses showed that, in general, simulation sessions happened in isolation from the rest of the curriculum and that there was lack of planned collaboration with the curriculum planning committee to ensure that pre-requisite knowledge is completed prior to the session. Student responses confirmed the lack of prerequisite knowledge acquisition prior to the simulation sessions.

The absence of completion of pre-requisite knowledge prior to the simulation activity is a significant concern. Knowing where content is taught is critical to curriculum leadership and to individual teaching faculty and students. A major challenge in an integrated curriculum that is taught by interdisciplinary faculty is presenting content with appropriate sequencing and layering to facilitate learning ( Sweller, van Merriënboer and Paas, 2019).

Best practices in curriculum design and simulation emphasize the importance of well-articulated learner outcomes and clear connection to course objectives as well as collaboration with faculty in planning and evaluation of each session. Educational strategies enforce the presence of a system to monitor the curriculum for congruence of objectives, methods, and assessments, sequencing, and coordination of content, and vertical and horizontal integration ( Sweller, van Merriënboer and Paas, 2019). However, curriculum delivery does not usually proceed in sequence, one step at a time, rather it is a dynamic and interactive process where curricular sequencing in general is constantly changing ( Thomas *et al.,* 2015). If neglected, this has some negative unintended consequences on learning outcomes and resulting in cognitive overload as described earlier.

As simulation-based education is being used more in the undergraduate curriculum, it is more important for the simulation activities to be purposefully planned and placed into the curriculum taking into consideration prerequisite knowledge.

An outcome-based curriculum identifies the objectives and learning outcomes of that session. Learning objectives should be related to clinical competence. However, it is important, again, to recognize that many objectives encompass more than one domain, and that there are enabling objectives necessary to achieve this ultimate objective and these require the attention of the curriculum developer and the assessor.

With simulation scenarios, it is important to involve faculty from across the curriculum from the planning stages. It would also be useful to involve educators at the level of curriculum design and those who deliver simulation to work together to discuss the enabling objectives and any potential barriers to progress as well as possible solutions ( Dent, Hardenand Hunt, 2007; Thomas *et al.,* 2015).With the emerging use of competency based medical education, educational curriculum must be planned carefully using varied clinical rotations and assessments ( Arora *et al.,* 2020). This standard applies to planning simulation activities.

## Conclusion

Proper and careful integration of simulation-based activities within the curriculum is particularly important in health professions education to ensure proper learning and avoid cognitive overload. This study sets the groundwork for larger-scale approaches to explore curriculum sequencing with simulation-based education. Best practices from the education literature can support this process. Future studies and next steps for development include examining better ways to integrate simulation-based activities into the curriculum through ensuring that prerequisite knowledge is completed prior to simulation activities. This should be actively sought and collaboratively planned by the simulation team and the stakeholders to achieve the intended learning outcomes without cognitive overload. Meeting the learning outcomes efficiently is the first step to producing efficient providers who will practice high quality care which in turn will improve patient safety. As we move to competency based medical education, the process of continuously analyzing the curriculum map in order to determine where best to place simulation into the curricula needs to be continuously monitored, reviewed, and evaluated.

## Take Home Messages

• Using complex scenarios in simulation-based education is gaining momentum in undergraduate medical education.
• Planning complex simulation scenarios requires completion of prerequisite knowledge and enabling objectives prior to the simulation session.
• Missing the integration of pre-requisite knowledge leads to cognitive overload and inadequate learning.
• Involving clinical and didactic faculty as well as the curriculum committee during the planning stages of complex simulation scenario design ensures proper integration of pre-requisite knowledge.
• Mapping the curriculum continuously will help ensure the right placement for simulation sessions within the curriculum.

## Notes On Contributors

Prof Dima Abdelmannan is an Vice Dean- Clinical Affairs and Adjunct Professor of Medicine at Dubai Medical College, Consultant Endocrinologist, Dubai Diabetes Center, Dubai Health Authority, Dubai, United Arab Emirates.
