# Supplementary material for: A pilot study to understand faculty and student needs for undergraduate medical education simulation sequencing in the United Arab Emirates
Source: MedEdPublish (2016). 2021 May 14;10:127. [Version 1] doi: 10.15694/mep.2021.000127.1 (PMC10939532; doi:10.15694/mep.2021.000127.1)
Supplement: Peer review history

# Reviewer report: Reviewer response for version 1

**DOI:** 10.21956/mep.18975.r27182

**Published:** 2021-07-19

**Authors:** Ken Masters

**Affiliations:** 1 Sultan Qaboos University

This review has been migrated. The reviewer awarded 3 stars out of 5 A small, but interesting, pilot study designed to understand faculty and student needs for undergraduate medical education simulation sequencing in the UAE. The Introduction is well-written, giving a succinct account of the current trends and thought and gaps in the literature. Overall, the study has been well-conducted, and reasonable conclusions are drawn. A few issues need to be addressed:• A little more detail about the online survey is required. o How, and by whom, was/were the survey form/s constructed?o The author says that the survey was sent to faculty; does this mean a link was sent? If so, which system was used? If not, how was anonymity retained? o The date range of the survey should also be given.o The first paragraph of the Result should go into the Methods. If any software was used in the theming, this should be identified.o Finally, it would be useful if the actual survey form/s could be given. • The Tables of data should be placed closer to where they are first mentioned, so Table 1 should be placed above the first mention of Table 2.• The paper needs to note at least two limitations:o The small sample size, ando The theming was performed by only one person, with no form of data verification.So, an interesting read, but does need a little more work. I would like to see Version 2 of the paper in which these issues are addressed.

Reviewer Expertise:

NA

No decision status is available

# Reviewer report: Reviewer response for version 1

**DOI:** 10.21956/mep.18975.r27180

**Published:** 2021-07-17

**Authors:** Hossam Hamdy

**Affiliations:** 1 Gulf Medical University

This review has been migrated. The reviewer awarded 4 stars out of 5 This study reemphasizes the importance of planning and sequencing when introducing simulations in a medical curricula. How, when and its relation to the program outcome competencies. This study explored an important aspect related to the use of complex simulation scenarios in the clinical phase of medical curriculum and the importance of ‘prerequisite knowledge’ needed in order to make the simulation session more effective. This can influence the curriculum content, organization and sequence. The author indicated it is a pilot study which justifies the small accessible sample size of faculty and students. The results highlighted the importance of students’ prior knowledge and communication between faculty during the planning and implementation of simulation in the curriculum. The organizational cultures and the clinical environment are main factors for the successful implementation of simulation in any health professional education program. This is an interesting pilot study which the author can expand on it to a multicenter study with other medical and health professional schools in the region.

Reviewer Expertise:

NA

No decision status is available

# Reviewer report: Reviewer response for version 1

**DOI:** 10.21956/mep.18975.r27181

**Published:** 2021-07-14

**Authors:** Demian Szyld

This review has been migrated. The reviewer awarded 4 stars out of 5 This survey based study evaluates perceptions of faculty and students about the preparation for learning with immersive simulation in the UAE but I think the findings are generalizable. The article is clearly written and the results are consistent with prior literature. I recommend this article as a useful contribution to the field that should be read by those with an interest in the area.

Reviewer Expertise:

NA

No decision status is available
